# Incidence of Newborns with Down Syndrome and Factors Associated with Their Length of Hospital Stay in Hospital Pakar Universiti Sains Malaysia

**DOI:** 10.21315/mjms-10-2024-838

**Published:** 2025-08-30

**Authors:** Muhammad Zul Hilmi Muhammad Zain, Ariffin Nasir, Najib Majdi Yaacob, Nor Rosidah Ibrahim

**Affiliations:** 1 Department of Paediatrics, School of Medical Sciences, Universiti Sains Malaysia, Health Campus, Kelantan, Malaysia; 2 Department of Paediatrics, Hospital Pakar Universiti Sains Malaysia, Kelantan, Malaysia; 3 Biostatistics and Research Methodology Unit, School of Medical Sciences, Universiti Sains Malaysia, Health Campus, Kelantan, Malaysia

**Keywords:** Down syndrome, newborn, length of hospital stay

## Abstract

**Background:**

Down syndrome (DS) is the most common chromosomal disorder worldwide. This study aimed to analyse the incidence of newborns with DS in a tertiary centre in the East Coast of Peninsular Malaysia and determine the factors associated with the length of hospital stay among them.

**Methods:**

This is a retrospective study from 2017 to 2021 that recruited 41 newborns with DS. We analysed patients’ clinical characteristics, comorbidities and length of hospital stay. Kaplan-Meier survival and Cox regression analyses were used to determine the median length of hospital stay and factors affecting the length of hospital stay.

**Results:**

The incidences of newborns with DS for five consecutive years were between 0.52 and 1.39 in 1,000 live births. The gender was equally distributed and the majority were delivered from advanced maternal age with multiple antenatal histories. The majority had non-disjunction trisomy 21 and were born with multiple comorbidities. Median length of hospital stay (IQR) among newborns with DS is 24 days (17.7–30.3). Every newborn with pulmonary hypertension (adjusted hazard ratio [aHR]: 0.24; 95% CI 0.10, 0.58), feeding intolerance (aHR: 0.16, [95% CI 0.06, 0.54]) and who had been invasively ventilated (aHR: 0.13, [95% CI 0.03, 0.48]) is expected to have a prolonged hospital stay.

**Conclusion:**

The incidence of newborns with DS in Hospital Pakar Universiti Sains Malaysia is lower than other countries worldwide. We found that pulmonary hypertension, history of invasive ventilation and feeding intolerance are the main factors that affect the length of hospital stay for newborns with DS in our hospital.

## Introduction

Down syndrome (DS) is the most common chromosomal disorder worldwide (1). The incidence of DS varies across different countries, influenced by factors like maternal age distribution, access to prenatal screening, and cultural attitudes towards pregnancy and childbirth. In developed countries such as the United States and those in Western Europe, the incidence rate is reported to be 1 and 2.3 per 1,000 live births (2, 3). While in Asian countries, the rate is approximately 0.8 per 1,000 live births (4). In Malaysia, there is a paucity of data on the incidence of DS of which was conducted 40 years ago in the largest tertiary government hospital (5).

Based on cytogenetic analysis, trisomy 21 can be classified into three types: free trisomy 21 or non-disjunction, translocation trisomy 21, and mosaic trisomy 21 (4). Free trisomy 21 is the most common type, which accounts for about 95%; 2% to 4% is Robertsonian translocation, and 1% to 3% is mosaicism (4). DS is always associated with multiple medical problems, such as congenital heart disease, gastrointestinal defects, and hypothyroidism, which can increase the likelihood of infection and feeding problems. Therefore, this will cause a high probability for hospital admission, usually to intensive care, and require a longer admission duration (6). Congenital heart disease and pulmonary hypertension become the most important factors that cause newborns with DS to stay longer in the ward (3, 7).

Luckily, in the past decades, we have seen a substantial increase in the life expectancy of this population group as a result of early screening for every medical problem during the neonatal period as well as successful early surgical intervention. Having said that, newborns with DS usually require a longer hospital stay due to their comorbidities and complications. To the best of our knowledge, there were no studies that specifically defined the actual duration of long hospital stays. For example, Seither et al. (6) reported that the mean days of hospital stay were 23 to 35 days (inborn versus outborn). On the other hand, Martin et al. (3) reported that the median days of hospital stay were 4 to 26 days (postnatal care ward only versus NICU admission).

As we saw cases of newborns with DS are rising and occupying most of the chronic patients in our neonatal intensive care unit and neonatal ward, the initiative is made to see the current incidence in our set up, specifically in Kelantan and to look at the most common factors for them to have prolonged hospital admission.

From this study, we hope that we can have a guide in managing our newborns with DS, especially in counselling parents regarding their children’s prognosis and tackling the most common cause so that we can shorten their hospital stay and subsequently avoid hospital-associated complications. Since no similar study has been conducted in Malaysia or Southeast Asia, we hope this will serve as a pilot study for future research aimed at addressing issues related to the management of newborns with DS.

The study aims to determine the incidence of newborns with DS in Hospital Pakar Universiti Sains Malaysia (USM) and to determine the factors associated with the length of hospital stay among them.

## Methods

### Study Design

This study adopts a retrospective cohort study, and all newborns with DS were followed up (retrospectively) until discharge. The study cohort consisted of all neonates born in our hospital with clinical features of DS and admitted to our hospital’s neonatal intensive care unit between 1 January 2017 and 31 December 2021. Patients’ records were traced from the Record Unit, Hospital Pakar USM. The data were collected based on case notes or patients’ information sheets available from the unit. All participants who fulfilled the inclusion criteria were included in the study, as this is a purposive sampling, and all available patients were recruited. Those with incomplete or missing data of more than 30% of the information were excluded from the study.

### Sample Size

The sample size was calculated based on survival analysis in PS-software version 3.0. Median length of hospital stay for DS without pulmonary hypertension (m1) is 5 days, median length of hospital stay for DS with pulmonary hypertension (m2) is 26 days, the accrual period (A) is 1,826 days with the additional follow-up time (F) of 60 days, and the ratio between groups (m) is three. The calculated sample size, including anticipation of a 10% dropout, was 18 patients.

### Data Collection

The data were collected starting from birth until the patient was discharged from the ward. There will be no follow-up after discharge home. The patient was identified by clinical features of DS or confirmed with chromosomal analysis as stated in the operational definition. The factors were then identified from the proforma. The study’s outcomes are based on the event, which is discharge home, and time to event, which is the length of hospital stay starting from birth to discharge, for survival analysis.

Retrospective record reviews were collected using a standardised proforma sheet involving all paediatric patients who were diagnosed with DS and born in our hospital. Data collected include socio-demographic data (gender, race, parents’ occupations and total family income per month), anthropometric data (birth weight, length and head circumference), birth data (mode of delivery and Apgar score), maternal data (gestational age, maternal age, parity and medical problems), karyotyping results, details of comorbidities, ventilation history, and length of hospital stay or length of survival day. In our unit, all newborns with DS will be screened for congenital heart disease and pulmonary hypertension by trained medical personnel.

### Operational Definition

Congenital heart disease is defined as any gross structural abnormality of the heart or intrathoracic great vessels that is present at birth (8). Pulmonary hypertension is defined as the presence of pre- and post-ductal saturation discrepancies of more than 10% and confirmed with echocardiography. The diagnosis was made from an estimated right ventricular systolic pressure of ≥ 50% or other echocardiographic findings suggestive of pulmonary hypertension, such as septal flattening, a dilated main pulmonary artery, and a dilated right cardiac chamber in the absence of pulmonary stenosis (8). Invasive ventilation refers to a form of mechanical ventilation where a tube is inserted into the patient’s airway, typically through the mouth (endotracheal intubation) or trachea (tracheostomy), to assist or control breathing (9). Airway anomalies are congenital or acquired structural abnormalities in the airways (including the trachea, bronchi, or larynx) that can affect normal breathing. These abnormalities may cause obstruction, narrowing, or malformations in the airway, leading to breathing difficulties or other respiratory issues (10). Feeding intolerance is the inability to digest feedings, presented as gastric residual volume of more than 50% of the previous feeding volume, abdominal distension or emesis or both, and disruption of the patient’s feeding plan (11).

### Statistical Analysis

All statistical analysis was performed by Statistical Package for the Social Sciences (SPSS) version 26, and a significance level of *P* < 0.05 was considered statistically significant. Descriptive statistics were used to summarise the socio-demographic and clinical characteristics of the study population. Continuous variables were presented as mean ± standard deviation (SD) or median (interquartile range) for non-normally distributed data, while categorical variables were presented as frequencies and percentages.

The incidence was determined by dividing the total number of newborns with DS by the total number of live births multiplied by 1,000 and presented according to the year. Kaplan-Meier survival analysis was used to determine the median length of hospital stay. Cox regression analysis was used to determine the factors (independent variables) associated with length of hospital stay among newborns with DS (dependent variable). Univariable analysis was conducted first to screen the significant independent variables. Factors that are known as clinically significant were analysed using multiple Cox regression analyses. Cox regression analysis (*P*-value < 0.25) was then proceeded with forward stepwise multiple Cox Regression Analysis to decide the predictive value (hazard ratio) for length of hospital stay. The lower the hazard ratio means the longer the length of hospital stays, hence, the risk of being discharged will be lower.

## Results

In this study, 41 newborns with DS were recruited. The incidences for four consecutive years are less than 1 in 1,000 live births. The year 2021 recorded the highest incidence of newborns with DS, which is 1.39 in 1,000 live births. The details of the result are shown in Table 1.

The clinical characteristics of patients, maternal factors, and comorbidities were summarised in Table 2. The gender was equally distributed, with 48.8% boys and 51.2% girls. The maternal age ranged between 24 and 44 years old. The majority of newborns (68.3%) were delivered by mothers of advanced age. Half of the mothers (51.2%) were diagnosed with gestational diabetes mellitus, followed by anaemia in pregnancy (24.4%), hypertension, and obesity (14.6%). However, some mothers have multiple antenatal problems, and the most common is gestational diabetes mellitus with obesity. Most of them (70.7%) were born appropriate for gestational age, and only 29.3% were born as small for gestational age. The majority of patients were delivered via spontaneous vertex delivery (63.4%), while the rest were via Caesarean section (34.1%); 11 of them were born via emergency lower segment Caesarean section (EMLSCS). Only one baby was delivered via instrumental delivery (2.4%). Out of 41 newborns, two of them (4.9%) did not have chromosomal analysis as they succumbed before the appointment date for the chromosomal study. From karyotyping results, we can see that 90.2% of them showed a non-disjunction type of trisomy 21, while only 4.9% showed mosaicism. For comorbidities, the majority of newborns were diagnosed with cardiovascular problems (87.8%). The most common non-cyanotic heart lesions were patent ductus arteriosus (PDA), ventricular septal defect (VSD), atrial septal defect (ASD), and patent foramen ovale (PFO), while cyanotic heart lesions were coarctation of the aorta and Tetralogy of Fallot. Most newborns had more than one congenital heart lesion. Meanwhile, 39% of them had airway anomalies like laryngomalacia, subglottic stenosis, and chronic lung disease.

From our study, the shortest duration of hospital stay was six days, and the longest was 179 days. During admission to the neonatal ward, 85.4% developed sepsis, whether presumed sepsis (presence of risk factors that predisposed babies to infection) or clinical (babies who exhibited any signs/symptoms of sepsis), with only 7.3% yielding positive cultures from tracheal aspirate or blood. Only 12.1% of them developed haematological problems like transient abnormal myelopoiesis and myelodysplastic disorder. About 53.7% exhibit endocrine problem, which is hypothyroidism, gastrointestinal (41.5%), and respiratory (39%). Almost half of them (43.9%) developed pulmonary hypertension. Twenty-four per cent of newborns required surgical intervention, like laparotomy, cardiac intervention, such as balloon angioplasty, PDA ligation, and PDA device occlusion, as well as otolaryngology intervention, like aryepiglottic fold release and tracheostomy during admission.

**Figure 1:**
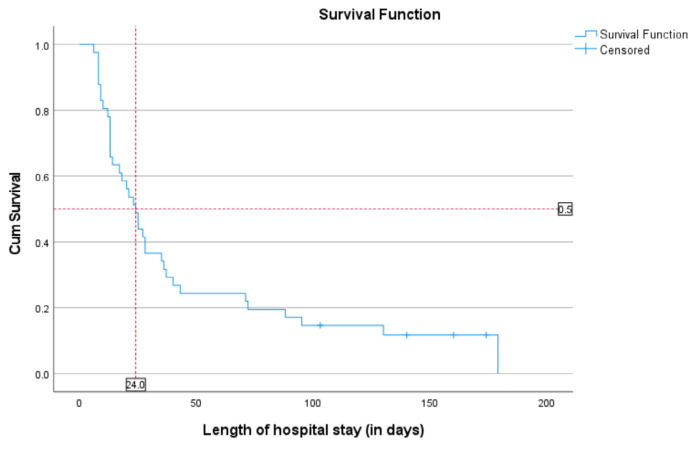
Kaplan-Meier survival curve in newborns with DS in relation to length of hospital stay The median length of hospital stay (IQR) 24 (95% CI: 17.7 to 30.3 days)

The Kaplan-Meier survival curve is used to determine the median length of hospital stay among newborns with DS. The median length of hospital stay is 24 days (Figure 1). Therefore, if the newborn is not discharged after 24 days, they will be categorised as having a longer hospital stay.

Table 3 describes the results of the univariate Cox proportional hazard model and identifies the factors associated with the length of hospital stay. The result showed congenital heart disease (HR = 0.55 [95% CI 0.21, 1.45], *P* = 0.229), pulmonary hypertension (HR = 0.37 [95% CI 0.19, 0.75], *P* = 0.005), invasive ventilation (HR = 0.11 [95% CI 0.04, 0.34], *P* < 0.001), airway anomalies (HR = 0.38 [95% CI 0.18, 0.79], *P* = 0.009) and feeding intolerance (HR = 0.8 [95% CI 0.03, 0.23], *P* < 0.001) are the significant factors for longer hospital stay. These five significant factors were further analysed using multivariate Cox regression. Every newborn with pulmonary hypertension (adjusted HR [aHR] = 0.24 [95% CI 0.10, 0.58], *P* = 0.001), feeding intolerance (aHR = 0.16 [95% CI 0.06, 0.54], *P* = 0.002) and who had been invasively ventilated (aHR=0.13 [95% CI 0.03, 0.48], *P* = 0.002), is expected to decrease the chance to be discharged home while congenital heart disease and airway anomalies are not an effective indicator for length of hospital stay.

Of 41 newborns, two passed away during their stay in neonatal intensive care. One baby passed away due to complications of congenital heart disease, while the other one passed away due to sepsis with positive growth from blood culture and tracheal aspirate.

## Discussion

The incidence of newborns with DS in Hospital Pakar USM is less than 1 in 1,000 live births for four consecutive years from 2017 to 2020, except in 2021, where it was reported as 1.39 in 1,000 live births. It is considered lower than other countries worldwide. This study only involved a single-centre. In addition, we only capture data for those born in our hospital. If the data is combined with another tertiary centre, the incidence might be comparable. India reported their incidence, which is almost comparable to our study (4). The last major study on DS in our country was conducted nearly 40 years ago at Hospital Kuala Lumpur by T S Hoe et al. (5) in 1989, which reported an incidence rate of 1 in 959 live births. More recently, a study by Zahari et al. (8) in 2019 focused specifically on DS cases that included congenital heart disease, as this condition significantly influences morbidity and mortality outcomes.

In this study, we examined the demographic, antenatal history, and clinical characteristics of newborns with DS and their length of hospital stay. From our data, almost 95% of them had non-disjunction trisomy 21, while the rest had mosaicism patterns. We could not capture any patient with translocation trisomy 21. Karyotyping for two newborns was not sent as they succumbed before the investigation was done, which might have the potential to be translocation trisomy 21.

A study by Mann et al. (12) in 2016, comparing DS newborns with unaffected newborns, found that those with DS regardless of whether they were diagnosed with congenital heart disease or not, will stay longer, had more time on oxygen, spent longer on special care and were discharged with home oxygen more frequently than unaffected newborn (11 days versus 5 days) (6). Meanwhile, a study by Martin et al. (3) in 2018 compared newborns with DS into three admission groups. Most of them have had NICU admission and have prolonged hospital stays. The majority of them were diagnosed with either pulmonary hypertension, gastrointestinal morbidities, or had a history of invasive ventilation. From these two studies, we identified the significant factors affecting their length of hospital stay were gender, birth weight, congenital heart disease, pulmonary hypertension, ventilation history, airway anomalies, and feeding intolerance. Our findings revealed that only pulmonary hypertension, ventilation history, and feeding intolerance show statistically significant effects on length of hospital stay among newborns with DS. In addition to a genetic predisposition from trisomy 21, pulmonary hypertension in DS may result from congenital heart disease (CHD), developmental lung disease, and airway obstruction. These factors contribute to the complexity of managing pulmonary hypertension in individuals with DS and highlight the need for comprehensive care approaches (8). The presence of pulmonary hypertension, regardless of its cause, is significantly associated with an increased need for ICU care (4). Like other normal newborns, any invasive ventilation will cause babies to stay longer as they may need longer oxygen support following extubation. A study by Seither et al. (6) in 2021, aside from cardiac conditions, another common medical reason for ICU admission is feeding intolerance (62%), in which eight of them required insertion of gastrostomy tubes. Feeding intolerance in newborns with DS can stem from several factors. The first one is sucking and swallowing incoordination. Due to hypotonia, they often struggle with coordinating their sucking and swallowing reflexes, making feeding difficult. Based on study by Stanley et al. (13) in 2019, those infants with feeding difficulties warranting referral for a Video fluoroscopic Swallow Study (VFSS), 55% of infants were found to have some degree of dysphagia (difficulty in swallowing) affecting the oral and/or pharyngeal phase while 39% had severe dysphagia requiring adjustments to breast milk/formula consistency or the need for non-oral feeding methods (13). Secondly, comorbidities such as airway and respiratory abnormalities significantly increased the risk of dysphagia and led to feeding problems. There will be a higher risk of developing feeding intolerance if they demonstrate desaturation with feeds (13). The third factor is anatomical abnormalities. Issues such as duodenal atresia can necessitate that the infant remain nil by mouth until surgical correction can be performed. Post-surgery, feeding is typically reintroduced gradually (14). Addressing these challenges often requires a coordinated effort from neonatologists, paediatric surgeons, gastroenterologists, and feeding specialists to ensure optimal outcomes for the infant.

In our study, two newborns succumbed after three months of admission due to complications of complex cyanotic heart disease, and the patient developed multiorgan failure following sepsis. From this, we can see only 4.8% of the mortality rate among them, which occurs beyond the neonatal period. With the advancement in health facilities, life expectancy can be prolonged. A study done by Weijerman et al. (2) in 2008 showed their neonatal mortality is only 1.65% which was low, even though it is still considered high if compared to the reference population (0.36%). Improved surgical techniques and earlier interventions for CHD in infants with DS have significantly reduced infant mortality and morbidity (2).

DS is a common chromosomal abnormality among newborns worldwide. DS imposes a significant medical and social cost globally. Understanding the burden and trends associated with DS, especially when analysed through the Socio-Demographic Index (SDI) and regional stratification, provides valuable insights for public health leaders, researchers, and clinicians (1). This information is crucial for developing targeted interventions, allocating resources effectively, and improving the quality of life for individuals with DS and their families. With the knowledge of common congenital abnormalities associated with this syndrome and complications caused by their muscle hypotonicity, we should anticipate those problems and the necessity for thorough screening before safely discharging them home, including karyotyping. Early detection will ensure early treatment and avoid further complications.

In summary, the incidence of newborns with DS in our hospital is relatively lower compared to other countries worldwide. Their median length of hospital stay is 24 days based on survival time. Among the seven factors that are known to influence the length of hospital stay, only three of them are significantly associated with longer hospital stay, which are newborns with pulmonary hypertension, history of invasive ventilation, as well as feeding intolerance.

Our study provides local data regarding newborns with DS, which is important in counselling parents regarding the prognosis, their expectations, and what they need to do to support the management of their babies. We hope a bigger scale of study involving other hospitals in Malaysia can be done so that it will reflect a clearer picture and burden in treating this special group of neonates. Towards the end, we are aiming for the best approach in minimising their morbidity and mortality rates.

To the best of our knowledge, this is probably the only research that explores the factors contributing to the length of hospital stay in newborns with DS.

### Limitations of Study

Our study has a few limitations. This was a single-centre study. Even though Hospital Pakar USM is one of the tertiary hospitals, most of the DS babies were born outside and were only referred after delivery. As this study only focused on inborn babies, there will be many dropouts as they did not fulfil the inclusion criteria. The relatively small sample size may not accurately represent the population of newborns with DS in our country. To obtain a more accurate representation in future research, we may need to conduct multicentre studies in collaboration with other hospitals across the state. This approach would increase sample size, enhance generalizability, reduce bias, and facilitate comprehensive analysis. Future studies should address these limitations to strengthen the evidence base and improve the care and outcomes for newborns with DS.

## Conclusion

Pulmonary hypertension, history of invasive ventilation, and feeding intolerance are the main factors for longer hospital stay in newborns with DS.

## Figures and Tables

**Table 1:** The incidence of newborns with DS in 1,000 live births in Hospital Pakar USM from 2017 to 2021

Year	Total number of live births	Total number of newborns with DS	Incidence
2017	9,154	7	0.76
2018	9,498	8	0.84
2019	9,437	5	0.52
2020	9,367	8	0.85
2021	9,346	13	1.39

DS = Down syndrome

**Table 2:** Patient characteristics and comorbidities (*n* = 41)

Variables	*n* (%)
**Gender**	
Boy	20 (48.8)
Girl	21 (51.2)
**Maternal age**	
< 35	13 (51.2)
≥ 35	28 (68.3)
**Antenatal illness** *	
GDM	21 (51.2)
Hypertension	6 (14.6)
Anaemia in pregnancy	10 (24.4)
Respiratory problem	3 (7.3)
Obesity	6 (14.6)
Renal problem	2 (4.9)
Hyperthyroidism	1 (2.4)
**Birth weight**	
SGA	12 (29.3)
AGA	29 (70.7)
**Modes of delivery**	
Normal	26 (63.4)
Instrumental	1(2.4)
Caesarean	14 (34.1)
**Karyotyping**	
Non-disjunction	37 (90.2)
Mosaicism	2 (4.9)
Not sent	2 (4.9)
**Pulmonary hypertension**	
Yes	18 (43.9)
No	23 (56.1)

SGA = small for gestational age; AGA = appropriate for gestational age;

* The total of antenatal illness in mothers may exceed 41

**Table 3:** Factors associated with the length of hospital stay in newborns with DS admitted to the neonatal ward

Factors	b	Crude HR (95% CI)	*P*-value	Adjusted b	Adjusted HR (95% CI)	*P*-value
Gender	−0.07	0.93 (0.48–1.80)	0.832			
Birth weight	−0.07	0.93 (0.46–1.91)	0.851			
Congenital heart disease	−0.59	0.55 (0.21–1.45)	0.229	−0.49	0.61 (0.21–1.76)	0.365
Pulmonary hypertension	−0.99	0.37 (0.19–0.75)	0.005	−1.42	0.24 (0.10–0.58)	0.001
Invasive ventilation	−2.17	0.11 (0.04–0.34)	< 0.001	−2.07	0.13 (0.03–0.48)	0.002
Airway anomalies	−0.98	0.38 (0.18–0.79)	0.009	−0.06	0.94 (0.42–2.10)	0.883
Feeding intolerance	−2.49	0.08 0.03–0.23)	< 0.001	−1.75	0.16 (0.06–0.54)	0.002

b = estimated regression coefficient; SE = standard error of estimated regression coefficient; HR = hazard ratio; no significant interaction between all significant factors in univariate Cox regression
